# Cucurbitacin D Induces G2/M Phase Arrest and Apoptosis via the ROS/p38 Pathway in Capan-1 Pancreatic Cancer Cell Line

**DOI:** 10.1155/2020/6571674

**Published:** 2020-09-22

**Authors:** Myeong-Sun Kim, Kangwook Lee, Jin Mo Ku, Yu-Jeong Choi, Kyungyul Mok, Doori Kim, Chunhoo Cheon, Seong-Gyu Ko

**Affiliations:** ^1^Department of Cancer Preventive Material Development, College of Korean Medicine, Graduate School, Kyung Hee University, Seoul 02447, Republic of Korea; ^2^Institute of Safety and Effectiveness Evaluation for Korean Medicine, Seoul 02447, Republic of Korea; ^3^Department of Preventive Medicine, College of Korean Medicine, Kyung Hee University, Seoul 02447, Republic of Korea; ^4^Department of Science in Korean Medicine, College of Korean Medicine, Graduate School, Kyung Hee University, Seoul 02447, Republic of Korea; ^5^Department of Global Public Health and Korean Medicine Management, College of Korean Medicine, Graduate School, Kyung Hee University, Seoul 02447, Republic of Korea; ^6^Department of Korean Medicine, College of Korean Medicine, Graduate School, Kyung Hee University, Seoul 02447, Republic of Korea

## Abstract

Pancreatic cancer has a poor prognosis with a five-year survival rate of less than 10%. Moreover, chemotherapy is mostly rendered ineffective owing to chemotherapy resistance and cytotoxicity. Therefore, the development of effective therapeutic strategies and novel drugs against pancreatic cancer is an urgent need. Cucurbitacin D (CuD), a plant steroid derived from *Trichosanthes kirilowii*, is an anticancer agent effective against various cancer cell lines. However, the anticancer activity and molecular mechanism of CuD in pancreatic cancer remain unknown. Therefore, we aimed to investigate the anticancer activity and molecular mechanism of CuD in the human pancreatic cancer cell line, Capan-1. CuD induced cell cycle arrest at the G2/M phase, apoptosis, and reactive oxygen species generation in Capan-1 cell line. In addition, CuD induced the activation of the p38 MAPK signaling pathway that regulates apoptosis, which was also inhibited by *N*-acetyl-L-cysteine and the p38 inhibitor SB203580. These data suggest that CuD induces cell cycle arrest and apoptosis via the ROS/p38 pathway in Capan-1 pancreatic cancer cell line; hence, CuD is a promising candidate that should be explored further for its effectiveness as an anticancer agent against pancreatic cancer.

## 1. Introduction

Pancreatic cancer is the seventh leading cause of cancer-related deaths worldwide [1] with a poor prognosis and a 5-year survival rate of less than 10% [2]. Pancreatic cancer is asymptomatic at the early stage, and most patients are diagnosed at an advanced stage [3]. Surgical resection and chemotherapy are performed for treating pancreatic cancer. However, treatment is difficult owing to late detection of the cancer, chemotherapy resistance, and cytotoxicity [4]. Therefore, the development of new effective therapeutic strategies and novel drugs against pancreatic cancer are urgently needed.

Although chemotherapy affects cancer cells, several traditional chemotherapeutic agents injure normal cells and cause side effects, such as vomiting, neuropathy, diarrhea, hair loss, and rash [5]. However, phytochemicals are relatively less toxic than traditional chemotherapy [6]. Many well-known anticancer drugs, such as taxol, vinca alkaloids, camptothecin, and podophyllotoxins, are obtained from plants [7].

Such phytochemicals reportedly inhibit apoptosis and cause cell cycle arrest in cancer cells [8–10]. When DNA is damaged by UV irradiation, chemotherapeutic drugs, or other stimuli, cells undergo apoptotic cell death [11]. As apoptosis progresses, the cell undergoes shrinkage and forms blebs, the DNA and organelles are ruptured, and then phagocytosed by macrophages [12]. Cell cycle arrest is a phenomenon that prevents cell proliferation and division of defective cells; it mostly occurs at the G1/S or G2/M checkpoint [13]. G2/M cell cycle arrest is affected by the activity of CyclinB1-CDK1 complex. When the CyclinB1-CDK1 complex is deactivated by p21 inhibitor and inactive cdc25c protein, cell cycle is halted at the G2 check point [14]. Accordingly, the G2/M checkpoint prevents the mitosis of cells until the damaged DNA or defectively replicated DNA is repaired before being transferred to daughter cells [15, 16].

Furthermore, as another apoptotic trigger, reactive oxygen species (ROS) are a byproduct of normal cell metabolism, and their levels are regulated to maintain homeostasis [17]. Hence, the disruption of the balance of intracellular ROS level causes oxidative stress and DNA damage [18]. High level of ROS results in damage to cellular structure containing DNA, lipids, and proteins, resulting in the development of various diseases, such as cancer, diabetes, neurological degeneration, and aging [19–22]. In pancreatic cancer cells, ROS induce apoptosis and cell cycle arrest through activation of c-Jun N-terminal kinases (JNK) and p38 pathways [23].

**Figure 1:**
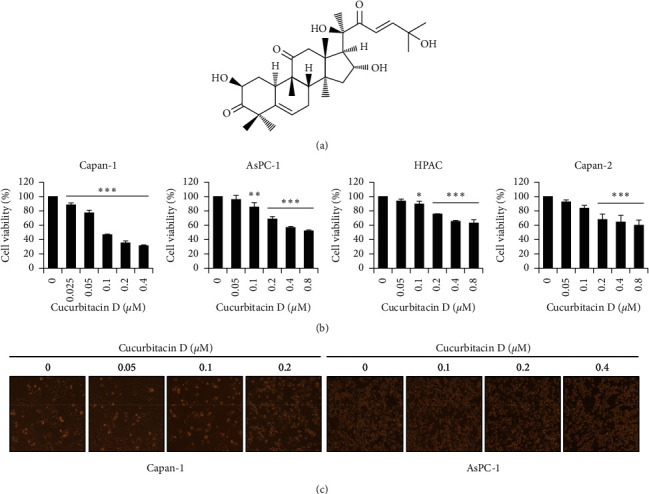
Cytotoxic effect of cucurbitacin D (CuD) on Capan-1 cell line: (a) chemical structure of CuD, (b) cell viability of pancreatic cancer cell lines upon exposure to CuD using the MTT assay. ^*∗*^*p* < 0.05, ^*∗∗*^*p* < 0.01, and ^*∗∗∗*^*p* < 0.001, significantly different as compared to the control, and (c) effect of CuD on Capan-1 and AsPC-1 cell morphology (bright-field image, 100x magnification).

An additional apoptotic inducer cucurbitacin D (CuD; Figure 1(a)), a member of the Cucurbitaceae family, is a plant steroid extracted from *Trichosanthes kirilowii*. CuD is reported to induce apoptosis in hepatocellular carcinoma and T-cell leukemia [24, 25]. Furthermore, CuD exhibits anticancer activity by inducing apoptosis and cell cycle arrest in human gastric, breast, cervical, ovarian, and lung cancers [26–30]. However, the anticancer effect of CuD and its underlying mechanism in pancreatic cancer have thus far remained unknown; hence, we aimed to investigate the effect of CuD on Capan-1 cell line.

## 2. Materials and Methods

### 2.1. Chemicals and Reagents

Roswell Park Memorial Institute (RPMI) 1640 Medium, Dulbecco's Modified Eagle's Medium (DMEM), penicillin/streptomycin, amphotericin B, and Trypsin-EDTA were purchased from Welgene (Seoul, Republic of Korea). Fetal bovine serum (FBS) was purchased from J R Scientific (Woodland, CA, USA). CuD was purchased from Extrasynthese (Genay, France). 3-(4,5-dimethylthiazol-2-yl)-2,5-diphenyltetrazolium bromide (MTT), propidium iodide (PI), 7-aminoactinomycin D (7-AAD), 2′,7′-dichlorofluorescein diacetate (DCF-DA), and *N*-acetyl-L-cysteine (NAC), SP600125 and SB203580, were purchased from Sigma-Aldrich (St. Louis, MO, USA). Annexin V was purchased from BD Biosciences (Franklin Lakes, NJ, USA). Primary antibodies against phospho-cdc2, phospho-25c, p21, cleaved caspase-7 and -8, cleaved PARP, JNK, c-jun, phospho-c-jun, p38, phospho-p38, and GAPDH were purchased from Cell Signaling Technology (Danvers, MA, USA). Primary antibodies against cyclin B1, cdc2, cdc25c, and phospho-JNK were purchased from Santa Cruz Biotechnology (Dallas, TX, USA). The secondary antibody, horse antimouse immunoglobulin G (IgG)-horseradish peroxidase (HRP) and goat antirabbit IgG-HRP were purchased from Cell Signaling Technology.

### 2.2. Cell Culture

Human pancreatic cancer cell lines Capan-1, AsPC-1, and Capan-2 were obtained from the Korean Cell Line Bank (Seoul, Republic of Korea). HPAC cell line was obtained from American Type Culture Collection (ATCC, Manassas, VA, USA). Capan-1, AsPC-1, and Capan-2 cells were cultured in an RPMI 1640 medium, and HPAC cell was cultured in a DMEM medium supplemented with 10% FBS, 100 U/ml penicillin, 100 *μ*g/ml streptomycin, and 250 ng/ml amphotericin B in a 5% CO_2_ humidified incubator at 37°C.

### 2.3. Cell Viability Assay

Cells (5 × 10^3^ cells/well) were seeded into a 96-well cell culture plate (SPL, Pocheon-si, Republic of Korea) with a culture medium. After overnight incubation, the cells were exposed to CuD at various concentrations (0.025, 0.5, 0.1, 0.2, 0.4, and 0.8 *μ*M) for 24 h. At the end of CuD treatment, 10 *μ*l of MTT solution (5 mg/ml) was added to each well and the plate was further incubated at 37°C in a CO_2_ incubator for 2 h. The formazan crystals were dissolved in 100 *μ*l of DMSO, and the absorbance was measured at 570 nm using a microplate reader (Molecular Devices, San Jose, CA, USA). The antioxidant agent, NAC, was added to suppress cellular ROS activity. Images of the cells were acquired using an inverted microscope (Olympus, Tokyo, Japan).

### 2.4. Cell Cycle Analysis

Cells were seeded into a 6-well cell culture plate at 5 × 10^5^ cells/well and then treated with CuD (0.05, 0.1, 0.2, and 0.4 *μ*M) for 24 h. A growth medium was added to 15 ml conical tubes, and then cells were harvested using Trypsin-EDTA followed by washing with DPBS (Welgene, Gyeongsan-si, Republic of Korea). After centrifugation (300 × g/4°C/10 min), the collected cells were fixed using 70% ethanol at 4°C for 30 min. The fixed cells were washed with DPBS and stained using the PI cocktail (50 *μ*g/ml PI and 50 *μ*g/ml RNase A) for 30 min at room temperature (20–25°C) in the dark. The cell cycle was analyzed using the FACSCalibur flow cytometer (BD Biosciences, Franklin Lakes, NJ, USA).

### 2.5. Apoptosis Assay

Capan-1 and AsPC-1 cells (5 × 10^5^ cells/well) were seeded in a 6-well cell culture plate and then treated with different concentrations of CuD (0.05, 0.1, 0.2, and 0.4 *μ*M). After 24 h, the cells were harvested, washed twice with DPBS, and stained with FITC Annexin V and 7-AAD for 15 min in the dark at room temperature (20–25°C). The extent of apoptosis was assessed using the FACSCalibur flow cytometer.

### 2.6. Measurement of ROS

The cellular ROS detection assay was performed using DCF-DA. Cells were seeded (5 × 10^5^ cells/well) into 6-well cell culture plates and incubated overnight. Subsequently, the cells were treated with various concentrations of CuD (0.05, 0.1, and 0.2 *μ*M) and 5 mM NAC for 24 h. The cells were then treated with 10 *μ*M DCF-DA for 30 min at 37°C. Finally, the cells were harvested, washed with DPBS, and measured for their ROS content using the FACSCalibur flow cytometer.

### 2.7. Western Blotting Analysis

To extract the protein, cells were lysed in the radioimmunoprecipitation assay (RIPA) buffer (Biosesang, Seongnam-si, Republic of Korea) for 30 min on ice and the cell lysate was centrifuged at 15,000 × g for 20 min at 4°C. The concentration of total protein in the collected supernatant was measured using the Bradford protein assay (Bio-Rad, Hercules, CA, USA). Equal amounts of protein (20 *μ*g) were loaded onto SDS-PAGE (10–12%) gels and electrophoresed for separation followed by transfer onto nitrocellulose membranes (GE Healthcare, Chicago, IL, USA). The membranes were blocked in 5% skim milk (BD Bioscience, Franklin Lakes, NJ, USA) in TBS-T (0.1% Tween 20 (Sigma-Aldrich, St. Louis, MO, USA)) buffer for 30 min and incubated with primary antibody at 4°C overnight. The membranes were then washed with TBS-T buffer and incubated with HRP-linked secondary antibody for 1 h at room temperature. Immobilon Western Chemiluminescent HRP Substrate (Merck Millipore, Burlington, MA, USA) was used for chemiluminescence detection.

### 2.8. Statistical Analysis

All data were analyzed using GraphPad Prism 5.0 (GraphPad Software Inc., San Diego, CA, USA), and the values are expressed as mean ± SD values. One-way ANOVA was used for determining the statistically significant differences between groups. *p* < 0.05 was considered statistically significant. All experiments were performed at least three times.

## 3. Results

### 3.1. CuD Inhibits Pancreatic Cancer Cell Line Viability

To examine the cytotoxic effect of CuD in the pancreatic cancer cell lines, we measured the cell viability by using an MTT assay. CuD significantly decreased the cell viability of pancreatic cancer cells compared to the untreated control (Figure 1(b)). In addition, microscopic images showed that CuD induces morphological changes, such as cell shrinkage and membrane blebbing, in Capan-1 and AsPC-1 cell lines (Figure 1(c)). These data suggest that CuD inhibits the cell viability of pancreatic cancer cell lines.

### 3.2. CuD Induces G2/M Cell Cycle Arrest in Capan-1 and AsPC-1 Cells

**Figure 2:**
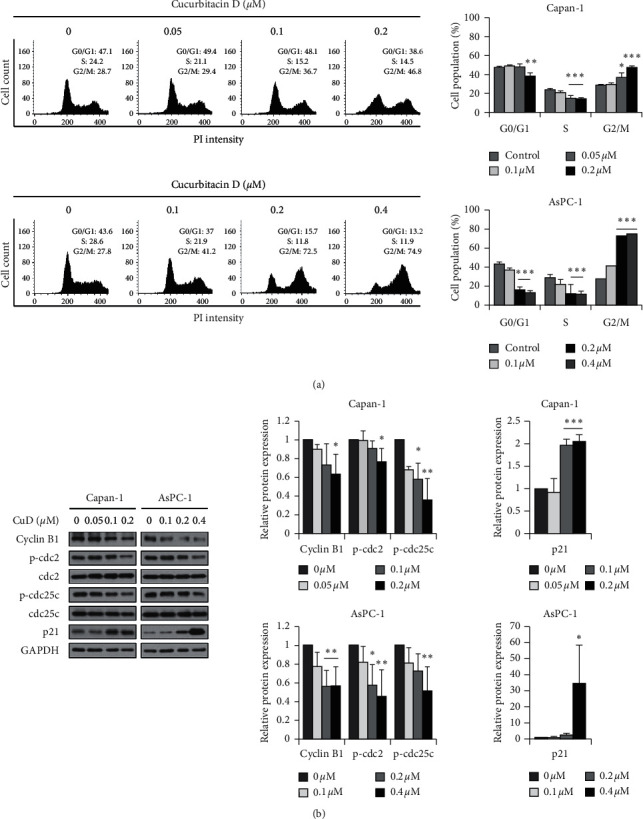
Cucurbitacin D (CuD) induces G2/M phase arrest in Capan-1 cell line: (a) CuD induces G2/M cell cycle arrest. The percentage of cell population in each phase is shown as mean ± SD from three independent experiments. ^*∗*^*p* < 0.05, ^*∗∗*^*p* < 0.01, and ^*∗∗∗*^*p* < 0.001, significantly different as compared to the control. (b) Capan-1 and AsPC-1 cells exposed to CuD for 24 h. The expression of G2/M cell cycle arrest markers was analyzed through western blotting. The histograms indicate the relative protein expression. Results are shown as mean ± SD from three independent experiments. ^*∗*^*p* < 0.05, ^*∗∗*^*p* < 0.01, and ^*∗∗∗*^*p* < 0.001, significantly different as compared to the control (0 *µ*M).

To investigate the inhibitory effect of CuD on the cell growth of Capan-1 and AsPC-1 cell lines, cell cycle distribution was analyzed. CuD increased the cell number at G2/M phase in a dose-dependent manner, accompanied by a reduction in the cell number at G0/G1 phase and S phase (Figure 2(a)). Western blotting analysis revealed that CuD downregulates cell cycle-regulating pathway markers, such as cyclin B1, phospho-cdc2, and phospho-cdc25c and upregulates the expression of cyclin-dependent kinase inhibitor, p21 (Figure 2(b)). Therefore, our data indicated that CuD induces G2/M phase cell cycle arrest in Capan-1 and AsPC-1 cell lines.

### 3.3. CuD Induces Apoptosis in Capan-1 Cells

**Figure 3:**
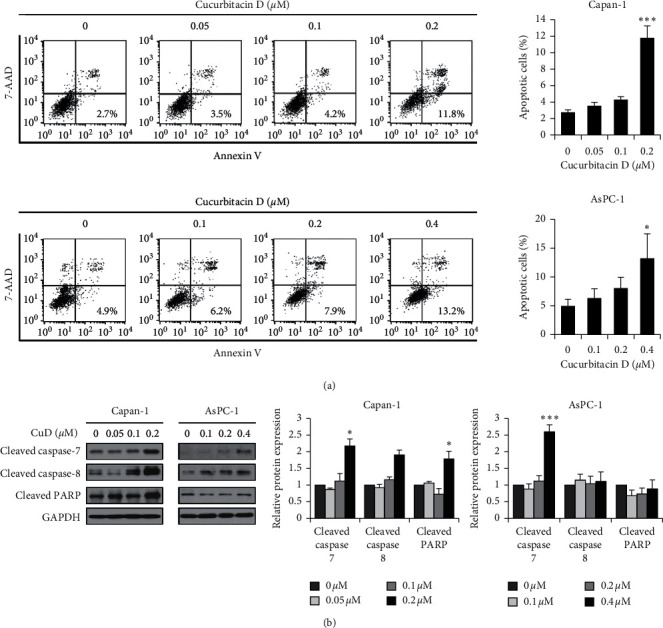
Induction of apoptosis by cucurbitacin D (CuD): (a) flow cytometry analysis of apoptosis in Capan-1 and AsPC-1 cells treated with CuD at a concentration of 0.05, 0.1, 0.2, and 0.4 *μ*M for 24 h; cells were stained with FITC Annexin V and 7-AAD and analyzed using flow cytometry. The histograms indicate the percentage of apoptotic cells. The percentage of apoptotic cells is presented as mean ± SD values from three independent experiments. ^*∗*^*p* < 0.05, significantly different as compared to the control. (b) Capan-1 and AsPC-1 cells incubated with CuD for 24 h; cell lysates were prepared and identified by western blotting for cleaved caspase-7 and -8 and cleaved PARP. The histograms indicate the relative protein expression. Results are shown as mean ± SD from three independent experiments. ^*∗*^*p* < 0.05 and ^*∗∗∗*^*p* < 0.001, significantly different as compared to the control (0 *μ*M).

We examined the effects of CuD on apoptosis in the Capan-1 cell line. CuD significantly increased the percentage of apoptotic cells compared to the control (Figure 3(a)). Thereafter, we performed western blotting to investigate the changes in apoptosis-related proteins and found that CuD upregulated cleaved caspase-7 and -8 and cleaved PARP in Capan-1 cell line, but only cleaved caspase-7 in AsPC-1 cell line (Figure 3(b)). Taken together, these findings suggested that CuD induces caspase-dependent apoptosis in Capan-1 cell line.

### 3.4. CuD Regulates Cell Cycle Arrest and Apoptosis by Inducing ROS Generation

**Figure 4:**
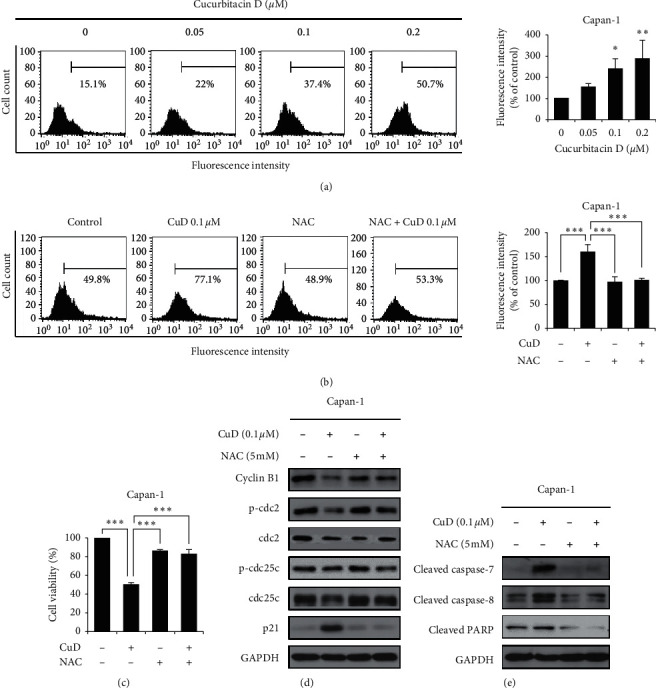
Reactive oxygen species (ROS) regulate cucurbitacin D (CuD)-induced cell cycle arrest and apoptosis: (a) cells were treated with various concentrations of CuD (0.05, 0.1, and 0.2 *μ*M) for 24 h followed by 10 *μ*M of 2′,7′-dichlorofluorescein diacetate (DCF-DA) for 30 min. The level of ROS was analyzed using flow cytometry. Fluorescence intensity is presented as mean ± SD from three independent experiments. ^*∗∗*^*p* < 0.01 and ^*∗∗∗*^*p* < 0.001, significantly different as compared to the control, (b) Capan-1 cells were preincubated with N-acetyl-L-cysteine (NAC; 5 mM) for 1 h and then treated with CuD (0.1 *μ*M) for 24 h. Fluorescence intensity is presented as mean ± SD values from three independent experiments. ^*∗∗∗*^*p* < 0.001, significantly different as compared to the control and NAC-treated groups, (c) cell viability was analyzed using the MTT assay in CuD-treated Capan-1 cells, pretreated with NAC (5 mM). Results are presented as mean ± SD values from three independent experiments. ^*∗∗∗*^*p* < 0.001, significantly different as compared to the control, and (d and e) western blotting analysis of G2/M cell cycle arrest-related proteins and apoptosis-related proteins in the CuD-treated Capan-1 cells, pretreated with NAC (5 mM).

We further examined whether CuD regulates cell cycle arrest and apoptosis by ROS generation in Capan-1 cell line. First, we evaluated ROS production by CuD in Capan-1 cells through flow cytometry. CuD significantly increased the fluorescence intensity of DCF (Figure 4(a)). Thereafter, we used the ROS inhibitor, NAC, to further prove the generation of ROS by CuD. Cotreatment with CuD and NAC effectively blocked CuD-induced ROS generation in Capan-1 cells (Figure 4(b)), while it markedly increased cell viability compared with CuD alone (Figure 4(c)). These data suggest that CuD induces cell death in Capan-1 cell line by inducing ROS production. Next, we investigated the regulation of cell cycle arrest and apoptosis by ROS generation using western blotting. When Capan-1 cells were cotreated with CuD and NAC, cyclin B1, phospho-cdc2, and phospho-cdc25c were upregulated and p21 was downregulated (Figure 4(d)). Moreover, NAC decreased CuD-mediated expression of cleaved caspase-7 and -8, and cleaved PARP (Figure 4(e)). Therefore, our data suggested that CuD-mediated ROS production is essential for the regulation of cell cycle arrest and apoptosis.

### 3.5. CuD Activates p38 via ROS Production

**Figure 5:**
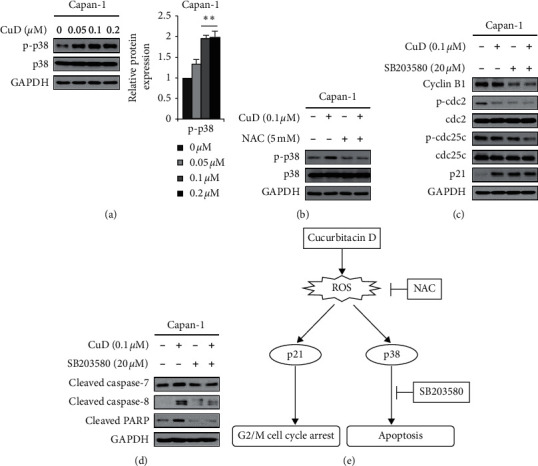
Cucurbitacin D (CuD) activates p38/c-Jun signaling pathway via generation of reactive oxygen species (ROS): (a) Capan-1 cells were treated with CuD (0.05, 0.1, and 0.2 *μ*M) for 24 h and activation of the p38 pathway was assessed via western blotting. The histograms indicate the relative protein expression. Results are shown as mean ± SD from three independent experiments. ^*∗∗*^*p* < 0.01, significantly different as compared to the control (0 *μ*M). (b) Cells were preincubated with *N*-acetyl-L-cysteine (NAC; 5 mM) for 1 h and then treated with CuD (0.1 *µ*M) for 24 h. Western blotting was performed to identify the ROS-mediated p38 signaling pathway in Capan-1 cells. (c and d) Capan-1 cell line was preincubated with SB203580 (20 *μ*M) for 1 h and then treated with CuD for 24 h. G2/M cell cycle arrest-related proteins and apoptosis-related proteins were analyzed by western blotting. (e) The schematic representation of the effects of CuD. CuD induces ROS generation, and then activates the p21 and p38 signaling pathways to induce G2/M cell cycle arrest and apoptosis, respectively.

As JNK and p38 MAPK signaling pathways are considered to play a crucial role in oxidative stress-induced apoptotic cell death [31], we determined the effect of CuD on JNK and p38 via western blotting analysis. CuD induced dose-dependent upregulation of phospho-p38 (Figure 5(a)) and phospho-c-Jun but did not alter phospho-JNK levels (Supplementary Figure S1(a)). Furthermore, cotreatment with CuD and NAC blocked the CuD-induced expression of phospho-p38 (Figure 5(b)) and phospho-c-Jun (Supplementary Figure S1(b)). Moreover, we treated the p38 inhibitor SB203580 and the JNK inhibitor SP600125 to confirm G2/M cell cycle arrest and apoptosis by p38 and JNK. Western blotting analysis demonstrated that although SB203580 did not affect G2/M cell cycle arrest, it decreased CuD-mediated expression of cleaved caspase-7 and -8 and cleaved PARP (Figures 5(c) and 5(d)). However, SP600125 induced G2/M cell cycle arrest and apoptosis rather than CuD (Supplementary Figures S1(c) and S1(d)). These data indicate that CuD-induced expression of p38 proteins is regulated through the production of ROS, resulting in apoptotic cell death of Capan-1 cells (Figure 5(e)).

## 4. Discussion

Pancreatic cancer has a very poor survival rate and is difficult to detect an early stage and difficult to treat [2–4]. Owing to the poor prognosis of pancreatic cancer owing to the ineffectiveness of current therapeutic approaches, the development of new anticancer drugs with fewer side effects is necessary. Phytochemicals have received increasing attention worldwide in recent years [32]. CuD is a common phytochemical derived from *Trichosanthes kirilowii*, a member of gourd family; its anticancer effects on various cancers have been identified [24]. However, it has thus far remained unknown whether CuD has an anticancer effect on pancreatic cancer. In this study, we analyzed the effect of CuD on Capan-1 pancreatic cancer cell line. The present results show that CuD induces G2/M cell cycle arrest and apoptotic cell death via the ROS-mediated p38 MAPK pathway in Capan-1 pancreatic cancer cell line.

Apoptosis and cell cycle arrest is triggered by DNA damage caused by ultraviolet light, alkylating compounds, biphenyls, and ionizing radiation [33]. Consequently, cells can prevent the transmission of defectively replicated DNA to the daughter cells [34]. Many phytochemicals have been known to induce apoptosis and cell cycle arrest in cancer cells [35, 36]. Cell cycle arrest and apoptosis induced by CuD have also been investigated in various cancer cell lines [27, 29]. This study shows that CuD induces cell cycle arrest and apoptosis in Capan-1 and AsPC-1 pancreatic cancer cell lines. Flow cytometric analysis confirmed that CuD increased the cell number in G2/M phase in Capan-1 and AsPC-1 cell lines. In addition, western blotting analysis revealed that CuD downregulates G2/M arrest-related protein markers such as cyclin B1, phospho-cdc2, and phospho-cdc25c and upregulates cyclin/CDK complex inhibitor p21 protein. These data indicate that CuD induces the G2/M phase cell cycle arrest. Flow cytometry revealed that apoptotic cells were increased following exposure to CuD. Moreover, CuD upregulated cleaved caspase-7 and -8 and cleaved PARP. These data indicate that CuD-induced apoptosis depends on the activation of caspases in pancreatic cancer cell lines.

ROS are maintained at a stable level intracellularly; however, high levels of ROS cause oxidative stress and damage to DNA, lipids, and proteins [19], resulting in apoptosis and cell cycle arrest [37]. Inducing ROS generation in cancer cells is recognized as a new strategy for cancer therapy [38, 39]. In a previous study, CuD induced ROS generation in gastric cancer [26]. This study shows CuD-induced generation of ROS on the Capan-1 pancreatic cancer cell line. Cotreatment with CuD and ROS inhibitor, NAC, reduced ROS generation and increased cell viability, and CuD-induced cell cycle arrest and apoptosis was inhibited. The present results show that the generation of intracellular ROS induces G2/M cell cycle arrest and apoptosis in Capan-1 cell line.

ROS reportedly activates the JNK and p38 MAPK signaling pathways [40]. JNK and p38 are activated by environmental stresses, such as oxidative stress, UV, DNA damage, and heat shock [41]. Activated JNK and p38 signaling induce cell cycle arrest and apoptosis [42, 43]. This study shows that CuD induced the phosphorylation of p38 and c-Jun. As confirmed via the use of the NAC, p38 and c-Jun phosphorylation was reportedly associated with ROS generation. However, no such effect was observed for JNK. Hence, we speculated that c-Jun could be activated by the other signaling pathways rather than the JNK pathway. JNK inhibitor SP600125 was used to confirm whether c-jun phosphorylation had an effect on cell cycle arrest and apoptosis, but there was no association with the CuD-induced G2/M cell cycle arrest and apoptosis. Rather, it induced the G2/M cell cycle arrest and apoptosis through a synergistic effect with CuD. In addition, the p38 inhibitor SB203580 did not increase cell proliferation-related protein expression when treated with CuD, but reduced apoptosis. It was confirmed that p38 phosphorylation was related with CuD-induced apoptosis. Together, these results suggest that CuD induces apoptosis via activating the p38 pathway through ROS generation in Capan-1 cell line.

## 5. Conclusions

In conclusion, this study shows that CuD induces G2/M cell cycle arrest and apoptosis via ROS-mediated activation of the ROS/p38 pathway in Capan-1 human pancreatic cancer cells. Further studies are required to explore the therapeutic effectiveness of CuD, a promising anticancer drug candidate.

## Data Availability

All datasets used and/or analyzed during the current study are available from the corresponding author on reasonable request.

## Supplementary Materials

Supplementary Materials Supplementary Figure 1: cucurbitacin D (CuD) activates the JNK signaling pathway via generation of reactive oxygen species (ROS). (a) Capan-1 cells were treated with CuD (0.05, 0.1, and 0.2 *μ*M) for 24 h, and activation of the c-jun was assessed via western blotting. The histograms indicate the relative protein expression. Results are shown as mean ± SD from three independent experiments. ^*∗*^*p* < 0.05, significantly different as compared to the control (0 *μ*M). (b) Cells were preincubated with *N*-acetyl-L-cysteine (NAC; 5 mM) for 1 h and then treated with CuD (0.1 *μ*M) for 24 h. Western blotting was performed to identify the ROS-mediated JNK signaling pathway in Capan-1 cells. (c and d) Capan-1 cell line was preincubated with SP600125 (20 *μ*M) for 1 h and then treated with CuD for 24 h. G2/M cell cycle arrest-related proteins and apoptosis-related proteins were analyzed by western blotting.
