# Supplementary material for: Cucurbitacin D Induces G2/M Phase Arrest and Apoptosis via the ROS/p38 Pathway in Capan-1 Pancreatic Cancer Cell Line
Source: Evid Based Complement Alternat Med. 2020 Sep 22;2020:6571674. doi: 10.1155/2020/6571674 (PMC7527894; doi:10.1155/2020/6571674)
Supplement: Supplementary Materials — Supplementary Figure 1: cucurbitacin D (CuD) activates the JNK signaling pathway via generation of reactive oxygen species (ROS). (a) Capan-1 cells were treated with CuD (0.05, 0.1, and 0.2 μM) for 24 h, and activation of the c-jun was assessed via western blotting. The histograms indicate the relative protein expression. Results are shown as mean ± SD from three independent experiments. ∗p < 0.05, significantly different as compared to the control (0 μM). (b) Cells were preincubated with N-acetyl-L-cysteine (NAC; 5 mM) for 1 h and then treated with CuD (0.1 μM) for 24 h. Western blotting was performed to identify the ROS-mediated JNK signaling pathway in Capan-1 cells. (c and d) Capan-1 cell line was preincubated with SP600125 (20 μM) for 1 h and then treated with CuD for 24 h. G2/M cell cycle arrest-related proteins and apoptosis-related proteins were analyzed by western blotting. [file 6571674.f1.docx]

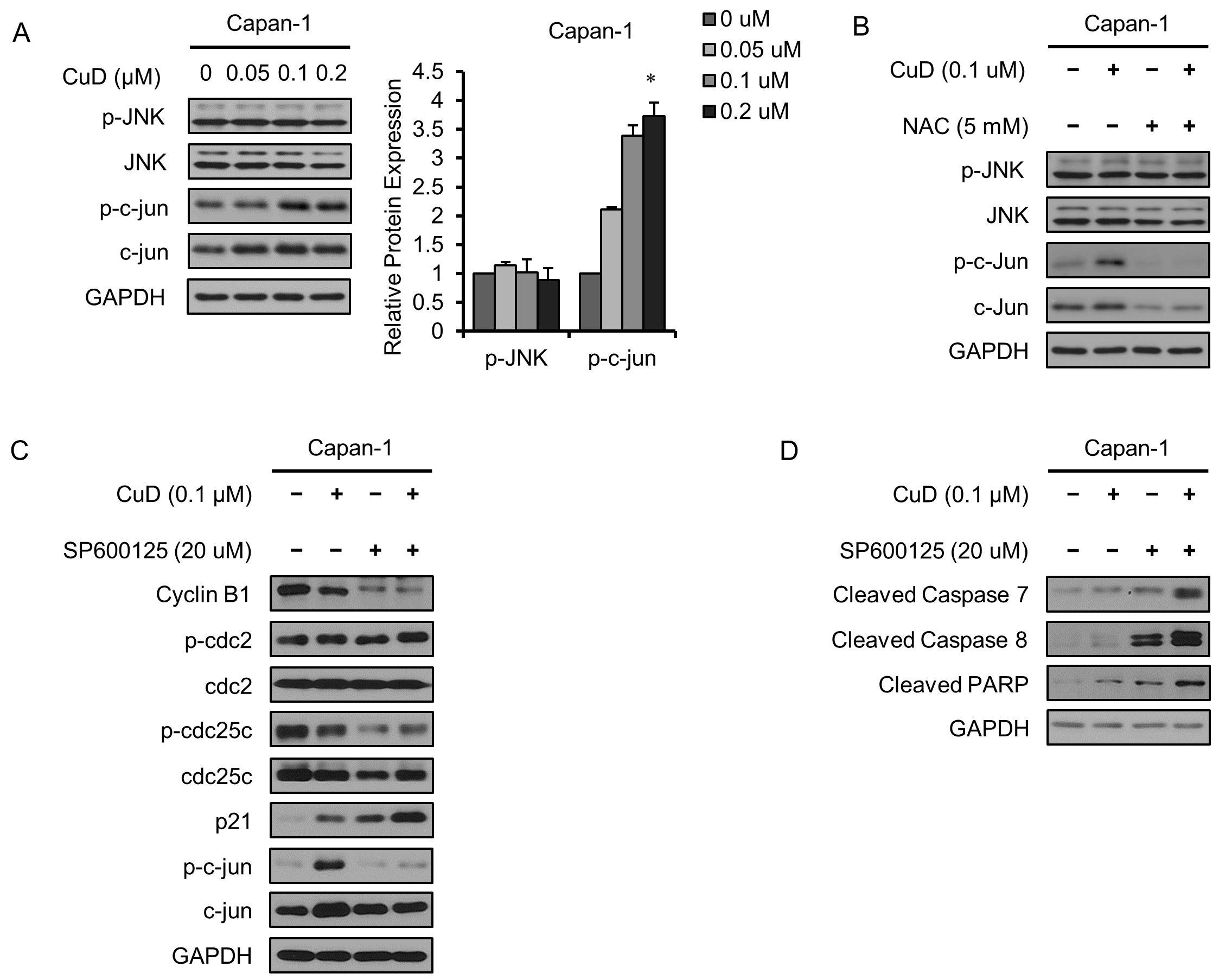


**Supplementary Figure 1. Cucurbitacin D (CuD) activates JNK signaling pathway via generation of reactive oxygen species (ROS).** (A) Capan-1 cells were treated with CuD (0.05, 0.1, and 0.2 µM) for 24 h, and activation of the c-jun was assessed via western blotting. The histograms indicate the relative protein expression. Result are shown as mean ± S.D. from three independent experiments. * *p* < 0.05, significantly different as compared to the control (0 µM). (B) Cells were preincubated with *N*-acetyl-L-cysteine (NAC; 5 mM) for 1 h, and then treated with CuD (0.1 µM) for 24 h. Western blotting was performed to identify the ROS-mediated JNK signaling pathway in Capan-1 cells. (C and D) Capan-1 cell line was preincubated with SP600125 (20 µM) for 1 h and then treated with CuD for 24 h. G2/M cell cycle arrest-related proteins and apoptosis-related proteins were analyzed by western blotting.
